# Improved IBD detection using incomplete haplotype information

**DOI:** 10.1186/1471-2156-11-58

**Published:** 2010-06-30

**Authors:** Giulio Genovese, Gregory Leibon, Martin R Pollak, Daniel N Rockmore

**Affiliations:** 1 Department of Mathematics, Dartmouth College, Hanover NH 03755, USA; 2 Renal Division, Department of Medicine, Brigham and Women's Hospital and Harvard Medical School, Boston MA 02115, USA; 3 Santa Fe Institute, Santa Fe, NM 87501, USA

## Abstract

**Background:**

The availability of high density genetic maps and genotyping platforms has transformed human genetic studies. The use of these platforms has enabled population-based genome-wide association studies. However, in inheritance-based studies, current methods do not take full advantage of the information present in such genotyping analyses.

**Results:**

In this paper we describe an improved method for identifying genetic regions shared identical-by-descent (IBD) from recent common ancestors. This method improves existing methods by taking advantage of phase information even if it is less than fully accurate or missing. We present an analysis of how using phase information increases the accuracy of IBD detection compared to using only genotype information.

**Conclusions:**

Our algorithm should have utility in a wide range of genetic studies that rely on identification of shared genetic material in large families or small populations.

## Background

Genetic studies designed to identify the location of loci that influence phenotypes depend on identifying regions of the genome that are shared among different individuals. This is true for both identification of rare, highly penetrant monogenic disease loci via linkage analysis or for common alleles that influence disease susceptibility via linkage disequilibrium as revealed by genome-wide association studies (GWAS). The use of very dense panels of single-nucleotide polymorphisms (SNPs) via microarrays makes direct identification of disease-associated variation possible in some study designs. However, in family-based studies of monogenic or oligogenic phenotypes, causal alleles are expected to have non-trivial penetrance and be relatively rare, thus making identification of disease-associated chromosomal regions a necessary prerequisite for identifying causal variation.

There has been tremendous recent progress at both ends of the spectrum for finding disease-influencing variants. There are useful techniques for identifying rare, but highly penetrant, monogenic disorders as well as for uncovering common variation conferring small but reproducibly increased risk. However, the middle ground of variants of moderate risk and moderate frequency is less well explored. Studies in large complex extended families and isolated populations with a high rate of specific phenotypes provide one method for approaching such phenotypes. Analyses of this sort would benefit from improved methods for identifying shared chromosome segments that rely neither on standard genetic linkage methods (which in turn rely on a near perfect understanding of family structure and are computationally very intensive) nor on association analysis (which is not well suited for identifying less common and more recent phenotype-influencing variants).

### Overview

Alleles that are identical on homologous chromosomes are said to be IBS (identical by state). IBS alleles are said to be IBD (identical by descent) if they are IBS by virtue of having been inherited from a recent common ancestor. It is common practice to identify chromosomal segments as "likely IBD" when a sequence of consecutive loci is observed to be IBS and is of such a length that the odds of this event happening by chance is small compared to the probability of that segment being inherited IBD. Thus, information about a series of consecutive loci informs the likelihood that alleles at any one of those loci are inherited IBD.

In the absence of informative parental genotypes at the loci of interest, the only absolute certainty as to whether a locus is IBD occurs when two genotyped individuals share no common alleles (say, one has genotype AB and the other CD). In this case no pair of autosomes is IBS, and we can conclude with certainty that the locus is not IBD. For bi-allelic markers, this can happen only when each individual is homozygous for a different allele (i.e., one individual is AA and the other is BB). Loci where this happens are said to be "incompatible" (for the pair) and, assuming no genotyping error has taken place, provide certainty for not being IBS and therefore not being IBD.

This simple observation provides the foundation for probabilistic approaches to identify likely IBD segments in [1] and [2]. These approaches consider regions IBD if no incompatible loci are observed on a sufficiently long segment. More intricate approaches using allele frequencies to weigh the evidence brought for IBD by every single locus are described in [3,4], and [5]. In [3] and [5] a Hidden Markov Model (HMM) is used in which there are two states, corresponding to being IBD and not being IBD, even though the process generating the states does not in general satisfy the Markov property. The model is chosen mainly for its relative simplicity and consequent computational tractability, as opposed to other approaches, such as those described in [6] and [7], which use inheritance vectors as states from which IBD status can be inferred. This inheritance vector approach is computationally intractable even for moderately sized pedigrees, but has the property that the underlying process is Markov.

Markov Chain Monte Carlo (MCMC) methods have been developed in [8] to deal with complex pedigrees, but they are not suitable for the common situation where information about the pedigree is inaccurate, incomplete, or spans only a few of the most recent generations.

Pedigree data can be useful for many purposes, but it does not in general provide a great deal of additional information for the purpose of IBD detection in situations in which the genotyping platforms are orders of magnitude finer than the expected number of recombination events in the pedigree. This can be the case when using Affymetrix or Illumina SNP microarrays. It is in fact possible to detect IBD segments and infer undetected relationships.

When haplotype (i.e., "phased" genotype) data is available, it is possible to identify as IBD segments that are shorter than those identified solely with genotype data. This is a key observation and we take advantage of this fact in the method we present below. Several algorithms use the haplotype to perform IBD detection [9] and association testing [10]. Note that even if we had the genome sequence data with base pair resolution, we would still be in need of a statistical model to infer haplotypes.

It is for these reasons that the "phasing" of genotype is important and relevant. Several different techniques have been used to infer the phase from genotype data in multiple individuals. Some techniques try to make such inferences by looking at thousands of individuals and then applying linkage disequilibrium (LD) to discern local haplotypes, using principles like maximum parsimony [11], entropy minimization [12], or variable length Markov chains [13]. These algorithms are fast and efficient. However, they require large sets of samples and while they tend to perform very well on the local scale, they perform less well, if at all, over long intervals. As a consequence, IBD detection algorithms using the inferred haplotype data end up identifying IBD segments broken up by short regions due to errors in the phase [9]. In general, assigning the most likely haplotype to a given sample without modeling the uncertainty can bias the results of studies based on haplotype.

An alternative approach to phasing is described in [14]. This technique uses IBD segments among relatives to infer the alleles that have been inherited from the maternal side and those that have been inherited from the paternal side of each individual. This approach is more robust than traditional methods and performs much better over the long range.

In this paper we introduce a new approach to the detection of IBD segments for a pair of samples. This approach uses the available phased genotype information to increase the accuracy of the detection, but at the same time is robust to inaccuracies of the phase. The method we present takes advantage of phase information even if this information is incomplete or less than fully accurate. In addition, we provide a novel method to improve the given configuration for the phased genotype through the identified IBD segments. We have implemented this algorithm and we detail its performance using genetic data in a large family known to be affected by an inherited form of kidney disease. In this example, our approach finds an additional 10% (probable) IBD loci as compared to the findings of an analogous method based only on genotype data. The ability to identify the entire set of regions that are actually IBD is of clear importance in identifying phenotype-influencing loci. Our algorithm should therefore be useful in a wide range of genetic studies that rely on the identification of shared genetic material.

## Results

**Figure 1:**
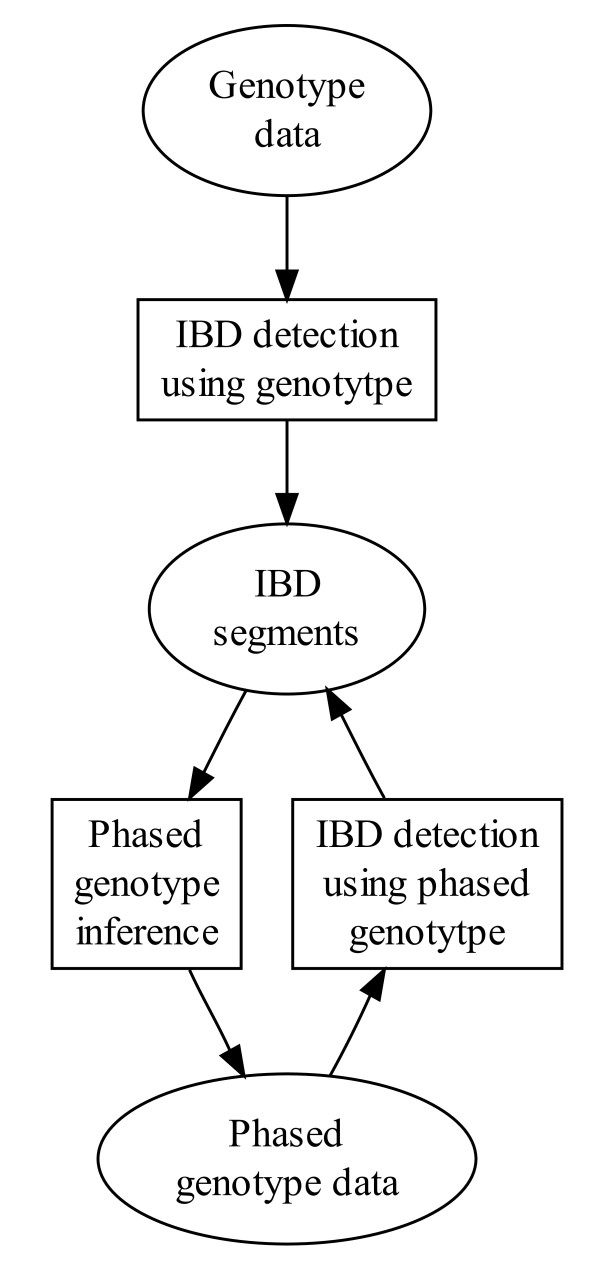
**Basic flowchart**. From genotyped data a first estimate of IBD segments is made. The IBD segments can then be used to estimate phase if this is missing, which in turn can be used to update (and improve) the estimate of IBD segments. The process can be repeated until it converges.

First we show a typical approach for detecting IBD segments from unphased genotype data, then we show how we can improve the detection algorithm by exploiting phase information, and finally we show how to use IBD information to update the phase. Figure 1 shows how the processes are integrated.

### IBD detection using genotype

Naively, a region would be identified as IBD for two samples if a long streak of loci for which at least one allele for one sample is IBS to one allele for the other sample is observed, while if the region is not shared IBD, we would expect to observe some loci as homozygous for both samples but for different alleles. Our approach to IBD detection expands on this basic idea and can be viewed as a descendant of the various HMM-based approaches. For context it is worth a quick review of the basic HMM approach. For a clear general explanation of HMMs see [[15], Chap. 3] and [[16], Section 3.10].

A typical HMM-based method for detecting IBD segments in a pair of samples uses a two-state model along two homologous pairs of autosomes belonging to two different samples (see e.g., [3]). We call this *HMM for genotype emission*. The two states correspond to the cases in which the two samples share at least one allele at a given locus (the state "IBD") and the case in which they do not share any allele by descent (the state "NO IBD"). It is possible to add a third state for the case in which both alleles are shared. It is also possible to identify seven additional unordered ways to be IBD between two samples (for a total of nine states), as described in [[17], Chap. 5] and introduced originally by [18]. This takes into account the possibility of more than a pair of segments being IBD among the four homologous chromosomes for the two samples. In order to mitigate the computational burden we decided not to consider this enlarged state space, although the extension to a greater number of states would be straightforward.

Because this model does not use phase information, the possible observed states can be partitioned into six groups. For bi-allelic markers, we will use the notation *A *and *B *to distinguish the two alleles. Because for every marker there is one version for each of two homologous chromosomes, we indicate the three possible (unordered) genotypes as *AA*, *AB*, and *BB*. Part A of Table 1 lists these states, together with the corresponding emission probabilities which depend on the allele probabilities in the population: *p *for allele *A *and *q *= 1 - *p *for allele *B*. These emission probabilities do not take into account the probability of genotyping error, which is an important variable to consider, as it is common to observe incompatible loci even when a pair of segments is clearly known to be IBD (for example in the case of small deletions where the apparent homozygous observation is actually caused by hemizygosity).

**Table 1:** HMM with genotype/haplotype emissions.

A	NO IBD	IBD	lev_*G*_
{AA, AA}	*p*^4^	*p*^3^	log_2_(*p*)
{AA, AB}	4*p*^3^*q*	2*p*^2^*q*	1 + log_2_(*p*)
{AA, BB}	2*p*^2^*q*^2^	0	*γ*
{AB, AB}	4*p*^2^*q*^2^	*pq*	2 + log_2_(*pq*)
{AB, BB}	4*pq*^3^	2*pq*^2^	1 + log_2_(*q*)
{BB, BB}	*q*^4^	*q*^3^	log_2_(*q*)

**B**	**NO IBD**	**IBD**	**lev_*H*_**

{A, A}	*p*^2^	*p*	log_2_(*p*)
{A, B}	2*pq*	0	*γ*
{B, B}	*q*^2^	*q*	log_2_(*q*)

Second and third column display emission probabilities for hidden states NO IBD and IBD for the HMM with genotype (A) and haplotype (B), with *p *the minor allele frequency, and *q *= 1 - *p*. The last column shows the log-evidence.

Note that while the property of IBD/NO IBD is not itself Markovian, the HMM framework still makes sense here: emission probabilities are defined in exactly the same manner, but initial and transition probabilities need a new interpretation. One way to proceed is by assigning these probabilities for each pair so that the defined Markov process has the correct equilibrium frequencies in accordance with the amount of genome shared IBD by the pair [3,5,9]. This way the choice of initial and transition probabilities are guided by the degree of relationship of the two samples being compared.

Our approach differs and instead regards the transition probabilities as *costs*. The smaller we keep the transition probabilities, the longer the streaks of compatible observations are needed to justify transitioning back and forth between the two states, and therefore the more likely it will be that we avoid false positive IBD detection. On the other hand, in doing this, we may incur false negative IBD detections. We show how to mitigate this and balance these considerations via the use of phase information.

For our purpose, the Markov chain is symmetric, with probability of transitioning between states ϵ = 2^-*δ*^, for a given positive *δ*, and with probability of remaining in the same state 1 - ϵ. There is an approximately linear relation between the variable *δ *and the length of the smallest IBD segment that can be detected by the model. Notice that our use of a single parameter contrasts with the approaches followed in [3] and [19], where the transitions probabilities are estimated in accordance with the expected number of IBD loci expected between the two samples and their expected length. Analogously, define initial probabilities as

Standard forward-backward decoding algorithms are then used to assign probabilities to the hidden variables, and each locus then is labeled as IBD if the probability of this event is greater than the probability of the opposite event.

One assumption of this analysis is that the observations for adjacent loci be independent of each other. This requires SNPs to be in linkage equilibrium. This is clearly not the case for SNPs in current genotype arrays, and relaxing this assumption would require a more sophisticated analysis. Nevertheless, this approach does still encode the intuition that haplotype data brings more evidence to avoid false positives than genotype data does.

In Appendix A we compare how easier it would be to detect IBD segments if we had haplotype information as opposed to genotype information.

### IBD detection using phased genotype

If we have the phased genotype information for each individual, then we could use a more detailed HMM, with one state representing the case in which no pair of autosomes is IBD and four different states each corresponding to the pair of autosomes (denoted as either "L" or "R") carrying the haplotype shared in the first and second sample. Following standard usage, when discussing *phased *genotype of heterozygous loci, we use the notation AB to indicate that allele A belongs to the "left" autosome and BA to indicate that allele A belongs to the "right" autosome. The definition of left and right autosome is of course purely arbitrary, but makes sense in the context of consecutive heterozygous loci. We will consider a choice for the phase for a group of heterozygous loci to be "correct" if it is consistent with the haplotypes of the segment containing those loci. In this situation the cases break down as

• IBD LL - The allele on the left autosome of sample 1 is IBD with the allele of the left autosome of sample 2,

• IBD LR - The allele on the left autosome of sample 1 is IBD with the allele of the right autosome of sample 2,

• IBD RL - The allele on the right autosome of sample 1 is IBD with the allele of the left autosome of the sample 2,

• IBD RR - The allele on the right autosome of sample 1 is IBD with the allele of the right autosome of the sample 2.

Up to ten additional states could be introduced to account for all possible cases (for a total of fifteen states), in which more than one pair of chromosomes are in an IBD state [[17], Chap. 5]. As before, for ease of exposition as well as computational efficiency we have chosen not to describe a model with this level of detail, but it would be straightforward to extend our techniques in this way.

**Figure 2:**
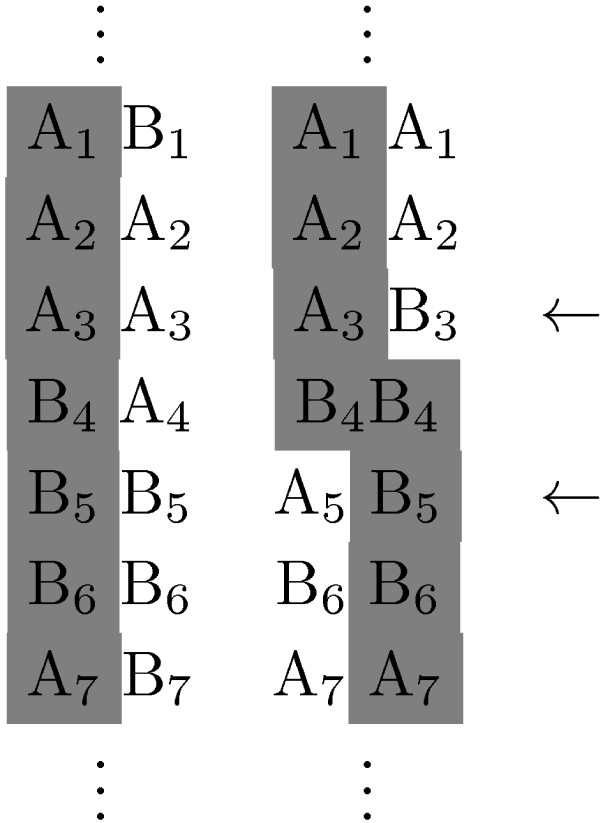
**IBD/haplotype inconsistency**. In this example, the two pairs of columns indicate phased genotype data. The shading shows a genotype consistency that gives evidence for an IBD region, and if this is the case, then the phasing of the second genotype is incorrect.

In the previous section we described how using haplotype information enables the identification of shorter IBD segments. Unfortunately, phased genotype data is not as easily available and, when it is, it is not always accurate. In some occasions it becomes clear that there are inconsistencies. For example we might encounter situations like the one in Figure 2 where we might recognize that one whole haplotype is shared, but only if the relative phase between the two loci indicated with the arrows is incorrect. On the other hand, on the level of genotype, this information would suggest that the region is IBD.

This suggests that it is of great importance for an IBD detection algorithm to be able to cope with uncertainty while at the same time still being able to exploit the available phase information. In order to account for the possibility of errors in the phase, the method we present here modifies the HMM by introducing transition probabilities between the four IBD states every time one of the observed loci is in a heterozygous state, so as to allow the tagged IBD segments to switch from one autosome to the homologous one.

We will call this new model *HMM with phased genotype emission*. The simple idea is that IBD segments should be allowed to switch between homologous autosomes at every heterozygous locus to allow for the possibility that the relative phase between two consecutive loci is incorrect.

**Figure 3:**
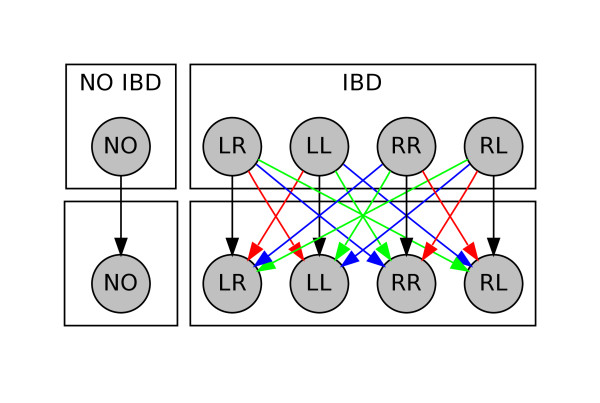
**Transition diagram for hidden states transitions**. Blue transitions are allowed only when the next genotype is heterozygous for the first sample, red transitions when the next genotype is heterozygous for the second sample, and green transitions when they are both heterozygous.

To achieve this, one of the four transition diagrams in Figure 3 (distinguished by the colors of their arrows) is used according to which genotype is observed next as shown in Table 2. Notice that if *λ *= *μ *= 0 then the four tables are exactly the same, which is equivalent to not allowing switching.

**Table 2:** HMM with phased genotype transitions.

A	NO	LL	LR	RL	RR	B	NO	LL	LR	RL	RR
NO		ϵ/4	ϵ/4	ϵ/4	ϵ/4	NO		ϵ/4	ϵ/4	ϵ/4	ϵ/4
LL	ϵ		0	0	0	LL	ϵ			0	0
LR	ϵ	0		0	0	LR	ϵ			0	0
RL	ϵ	0	0		0	RL	ϵ	0	0		
RR	ϵ	0	0	0		RR	ϵ	0	0		

**C**	**NO**	**LL**	**LR**	**RL**	**RR**	**D**	**NO**	**LL**	**LR**	**RL**	**RR**

NO		ϵ/4	ϵ/4	ϵ/4	ϵ/4	NO		ϵ/4	ϵ/4	ϵ/4	ϵ/4
LL	ϵ		0		0	LL	ϵ				
LR	ϵ	0		0		LR	ϵ				
RL	ϵ		0		0	RL	ϵ				
RR	ϵ	0		0		RR	ϵ				

The matrices show the transition probabilites among the five hidden states when the next genotype is respcectively homozygous for both samples (A), homozygous for the first sample and heterozygous for the second sample (B), heterozygous for the first sample and homozygous for the second sample (C), and heterozygous for both samples (D), with  = 1 - *ϵ*,  = 1 - *λ*, and  = 1 - *μ*.

The constants *λ *and *μ *are chosen according to how unlikely we expect that switching should take place. If the current phase configuration is completely random, then we would expect switching and not switching to be equally likely. If we model this by choosing *λ *= *μ *= 1/2, then the results are equivalent to those obtained using the HMM with genotype emission probabilities. We prove this in Appendix B. The method works well and does not run the risk of missing IBD segments that otherwise would be identified with the more simple HMM with genotype emission.

### Phased genotype inference from IBD segments

**Figure 4:**
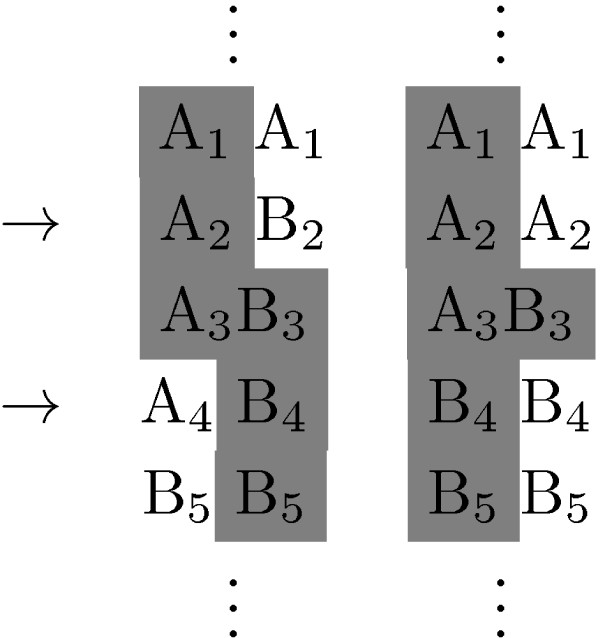
**Example of unsatisfied link**. The two heterozygous loci indicated with the arrows are said to be "linked" because they are contained in an IBD segment for which the other sample is homozygous and no other locus in between has the same property and the link is "unsatisfied" because the current phased genotype requires a switch in the IBD pattern.

Once a segment has been labeled as IBD between two pairs of autosomes, we know that at least one haplotype is shared. At a given polymorphic locus *i*, let *A*_*i *_denote the more common allele, and *Bi *the less common allele. In Figure 4 we see an example in which either haplotype *A*_1_*A*_2_*A*_3_*B*_4_*B*_5 _or haplotype *A*_1_*A*_2_*B*_3_*B*_4_*B*_5 _is shared, but we cannot infer with certainty which.

**Figure 5:**
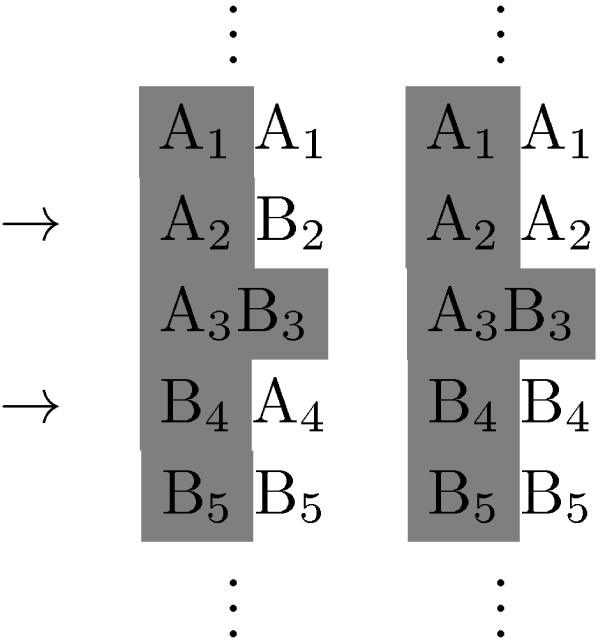
**Example of satisfied link**. The two heterozygous loci indicated with the arrows are said to be "linked" because they are contained in an IBD segment for which the other sample is homozygous and no other locus in between has the same property and the link is "satisfied" because consistent with the IBD segment detected.

However, even if we might not be sure exactly which haplotype is shared, we can usually rule out some configurations for the phased genotype and therefore we can still perform some level of inference. In this example the two loci indicated with the arrows are heterozygous for one sample and homozygous for the other sample and no other locus in between has the same property. In this case we say that the two heterozygous loci indicated with the arrows are "linked." The link is said to be "satisfied" if the phasing is such that the IBD region between them is consistent on the level of haplotype and "unsatisfied" if not (i.e., consistency between the linked loci is obtained by switching between autosomes). Thus the example above shows an "unsatisfied" link. Were this the original phasing of the genotype data, this could be remedied by a rearrangement of the first sample. Since the phased genotype was such that the two heterozygous loci were both on the left for the first sample, a possible solution would be to pick a configuration for the phased genotype that would look like the one in Figure 5.

We record a configuration like the previous one as a *link *between the two heterozygous loci. Once a sample has been compared with all the other available samples for shared IBD segments, the phased genotype that satisfies the largest number of links is computed.

**Figure 6:**
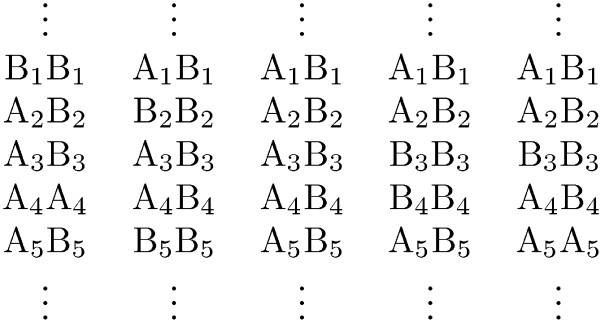
**Example of haplotype configuration before phasing**.

**Figure 7:**
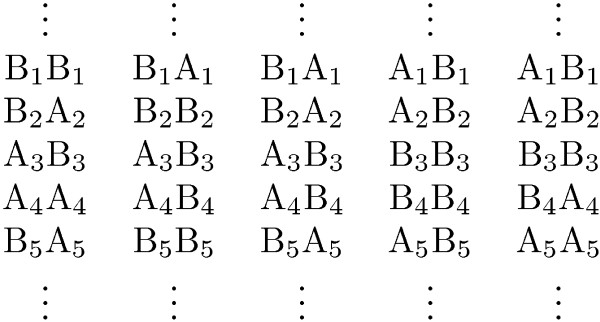
**Example of haplotype configuration after phasing**.

At this point it is important to notice that it is unlikely that all links could be satisfied, since it is usually the case that some might be due to genotype errors, false positive IBD segments, recombination events, or overlap of IBD segments on the two homologous chromosomes (as is common for siblings). Therefore we should aim at satisfying as many links as possible rather than trying to solve the likely impossible problem of satisfying them all. A priori, this combinatorial problem could get quite complicated. For example, consider the situation in Figure 6 with five samples for which the one in the middle is identified as IBD with all the other samples. In checking the different possible configurations, we end up concluding that the only configuration that is compatible with all the IBD segments detected is the one in Figure 7.

Because of errors, it will not be possible in general to find a configuration that is compatible with all IBD segments detected. Therefore we use a maximum satisfiability approach. For a given sample, *target*, let *t*_1_,....,*t*_*m *_denote the locations of the heterozygous loci. For every phasing configuration of the genotype, associate a binary vector (*y*_1_, *y*_2_,...,*y*_*m*_) with the convention that if *y*_*i *_= 0, then the phased genotype at locus *t*_*i *_is AB, otherwise if *y*_*i *_= 1 then the phased genotype is BA. Now, for each sample *target *we initialize a sparse square matrix *L*_*target*_. For each IBD segment sample *target *shares with another sample *source*, we search for all consecutive pairs of loci (*t*_*i*_, *t*_*j*_) for which sample *target *is heterozygous at both loci *t*_*i *_and *t*_*j *_while sample *source *is homozygous, and no other locus in between *t*_*i *_and *t*_*j *_has the same property.

For each pair (*t*_*i*_, *t*_*j*_) defined this way, we increment *L*_*target*_(*I*, *j*) by one if for sample *source *we observe either  and  or  and , and we decrement *L*_*target*_(*i*, *j*) by one if for sample *source *we observe either  and  or  and . Pseudocode is given in Algorithm 1 in Appendix C.

Once we update matrix *L*_*target *_using all the IBD segments shared with other samples, we are then interested in finding the binary vector (*y*_1_, *y*_2_,...,*y*_*m*_), for which the sum(1)

is as large as possible. In fact, notice that the expression in (1) is equal to the number of links satisfied by the phase configuration given by the binary vector (*y*_1_, *y*_2_,...,*y*_*m*_) minus the number of unsatisfied links. Moreover the number of loci (*t*_*i*_, *t*_*j*_) for which *L*_*target*_(*i*, *j*) is not zero is linear in *m *since the likelihood of finding a link between loci *t*_*i *_and *t*_*j *_decreases exponentially with the difference *j *- *i*.

The general problem is a special instance of the more general class of MAX GEN2SAT problems [20]. This a class of problems which try to find the configuration of a collection of binary variables with constraints on pairs of variables so to satisfy as many constraints as possible. It is known that for such problems it is possible to find a configuration that satisfies at least 87.856% of the maximum number of satisfiable links [21] in time polynomial in the number of binary variables.

However, a general algorithm is not so useful for our particular problem. On average we would expect links between *t*_*i *_and *t*_*j *_for *j *- *i *very small and most of the time when *j *- *i *= 1, as chances for a locus to be heterozygous for both samples are always lower than 50%. This still makes the two binary coefficients *y*_*i *_and *y*_*j *_highly dependent on each other, even if *j *- *i *is large, as each IBD segment will establish a chain of dependencies. A better approach would be to consider the relative binary phase coefficients

where ⊕ corresponds to the logical xor (the addition of numbers modulo 2). Using relative binary phase coefficients makes it easier to devise an algorithm that performs an iterative climb in the space of all possible combinations of binary phase coefficients. Testing with the FGFM dataset (see the Experiments section below) proved that very good results are already possible with a naive approach that will search among all possible configurations of *s *binary coefficients (*z*_*i*_, *z*_*i*+1_,...,*z*_*i*+*s*_) for the one that satisfies the largest number of links for *i *= 1,...,*m *- *s *- 1. Pseudocode is given in Algorithm 2 in Appendix C. Note that an important conceptual advantage of this approach is that each link provides information with respect to the relative phase of two heterozygous loci, usually close to each other, leading to a simpler approach to handling conflicting information coming from multiple IBD segments. In fact, trying to determine which alleles have been inherited from the maternal chromosomes and which have been inherited from the paternal chromosomes (as is done in [14]) might be more problematic when dealing with information coming from short IBD segments for which the inheritance pattern is unclear. A straightforward example comes from phasing the genotype of an individual using the genotype of a child. All loci will be easily recognized as IBD although we cannot infer where the recombination events took place. Nevertheless, only one link per recombination event would not be satisfied by the true haplotype. If no other evidence is available there is no way to infer the correct phase, but if other links are collected around the recombination event from other sources, it is likely that the true haplotype will be the one satisfying the maximum number of links. The same argument can be made for links not satisfiable by the true haplotype which are due to genotype errors in the source sample or to false positive IBD segments.

### Experiments

We performed two different analyses, the first one aimed at understanding how much phase information is concealed in the IBD segments and the second one aimed at quantifying how much IBD detection with phased genotype, even if incomplete, can be achieved and how this outperforms the one with genotype.

To compare how phasing using IBD segments compares with other haplotype phasing methods, we simulated the dynamics of a chromosome segment reproducing through a small population using a Wright-Fisher model with recombination events. For a clear description of this model see [[22], Chap. 3]. The *switch error rate *(see [13,23]) measures how often the relative phase between heterozygous loci has been retrieved correctly. As an example, suppose that a sample consists of two haplotypes A_1_A_2_A_3_B_4_B_5_A_6 _and B_1_A_2_B_3_B_4_A_5_B_6_, but the inferred haplotypes are instead A_1_A_2_B_3_B_4_A_5_B_6 _and B_1_A_2_A_3_B_4_B_5_A_6_. Then the switch error rate is 33%, since the relative phase between the first and third locus is incorrect, while the relative phases between the third and fifth locus and between the fifth and sixth locus are correct. The second and fourth loci do not matter since they are homozygous for the sample.

To indicate how much information is concealed in pairwise IBD information of a group, we compared the switch error rate of the haplotype retrieved by our phase update algorithm using IBD segments (as computed from the simulation) and how much relative phase was instead retrieved by the BEAGLE algorithm (see [13]). We also computed how much phase could be retrieved correctly by using both BEAGLE and the IBD segments.

To simulate a scenario similar to a large interbred family, we ran different simulations with a different set of parameters. We first simulated a population of 2*N *haploid haplotypes of genetic length *L *centimorgans containing *s *SNPs for *T *generations using the Wright-Fisher model. Then to resemble the dynamics of a small interbred family we sampled a subset of 2*n *haplotypes and we simulate them as a smaller population for *t *generations. To avoid having to model how SNPs arise in a population, we bootstrapped the whole simulation using haplotypes from the European population in the third phase of the Hapmap project http://hapmap.ncbi.nlm.nih.gov/, for which reliable phased data was computed using trios by a method similar to the one described in [24]. Results for different values of the parameters are given in Table 3.

**Table 3:** Comparison of switch error rates.

n	L	IBD	BGL	BOTH
50	10	3.88%	6.17%	2.41%
100	10	2.99%	4.54%	1.81%
200	10	1.96%	2.23%	1.18%
50	5	3.73%	4.87%	1.83%
100	5	2.13%	2.02%	1.01%
200	5	1.47%	1.01%	0.63%
50	2	3.81%	3.16%	1.57%
100	2	2.70%	1.20%	0.62%
200	2	1.55%	0.55%	0.35%

The table shows results for the switch error rate by, respectively, our phase update algorithm (see Appendix C for pseudocode) in the IBD column, the BEAGLE algorithm in the BGL column, and the combined approach in the BOTH column for a dataset simulated by using *N *= 10000 as the size of the original population, *T *= 100 as the number of generations for which the population was simulated, *n *as the size of the family sampled, *t *= 5 as the number of generations for which the family was simulated, *L *as the genetic length in centimorgans of the haplotypes simulated, and *s *= 400 as the number of SNPs in the haplotype.

These results are indicative of how much information is being missed by the BEAGLE algorithm and that could have still been retrieved using complete and correct IBD segment information. It is clear that BEAGLE scales very well as the size of the samples increases. However it is also clear that BEAGLE misses some of the correct switches which could be correctly inferred if the information from IBD segments was correctly exploited. This is true in particular for less dense SNP arrays. It is likely that while BEAGLE will perform very well when predicting the phase between closely linked markers, it might not do so for markers at larger genetic distance from each other, as could be the case for markers at opposite sides of a recombination hotspot.

From an IBD detection point of view, it is important to stress the fact that a switch error rate smaller than 10% is already very good for locating more IBD segments than with a pairwise IBD detection model solely based on genotype. So, when the parameters allow it, a good strategy is to bootstrap the analysis of IBD segments by first haplotyping the data using a haplotype phasing algorithm like BEAGLE. When this is not the case, for example if the markers are not dense enough or if not enough samples are available, our phasing algorithm can come in handy. A good strategy would be to alternate IBD detection and phase update in the small dataset, while decreasing the transition probabilities between IBD states at every step. In Table 4 the last four columns show the percentage of IBD regions (false negatives) missed by the IBD detection algorithm using only genotype data and the same quantity using complete haplotype data, together with the percentage of regions incorrectly identified as IBD (false positives) compared to the total amount of IBD regions, again using only genotype data and complete haplotype data respectively. Notice that when the size of the family sampled from the population *n *increases, the relative error detection rate increases as well. This is due to the fact in a larger family the average amount of IBD among different individuals is smaller, implying that the relative amount of regions shared IBD decreases.

**Table 4:** Comparison of IBD detection accuracy.

n	L	FN GT	FN HT	FP GT	FP HT
50	10	30%	9.9%	0.67%	0.29%
100	10	26%	13%	1.5%	0.65%
200	10	32%	28%	3.4%	0.75%
50	5	30%	2.9%	0.83%	0.19%
100	5	15%	5.6%	3.3%	0.85%
200	5	19%	13%	7.1%	1.6%
50	2	29%	2.6%	5.4%	4.8%
100	2	18%	2.4%	16%	14%
200	2	14%	5.5%	39%	34%

A constant size population of *N *= 10000 diploids of *L *centimorgans containing *s *= 400 SNPs was simulated for *T *= 100 generations using a Wright-Fisher model, then a family of size *n *was sampled and simulated as a small population for *t *= 5 generations. The last four columns measure, after performing IBD detection, the rate of false positives using genotype in the FN GT column, and using phased genotype in the FN HT column, and the rate of false positives using genotype in the FP GT column, and using phased genotype in the FP HT column. Parameter choices when using genotype were *γ *= 8, *δ *= 12, and *λ *= *μ *= 1*/*2, and when using phased genotype were *γ *= 8, *δ *= 32, and *λ *= *μ *= 1 = 256. Results were averaged over 10 different simulations per choice of parameters.

The percentages relate to the total amount of loci pairwise shared by descent from one of the founders used to bootstrap the simulation. Notice that if two founders shared a segment IBD, their two haplotypes would not have been counted as IBD from the simulation, but they might have been detected as IBD by the IBD detection algorithm if inherited by two different samples, resulting in a false positive.

### FGFM family

**Figure 8:**
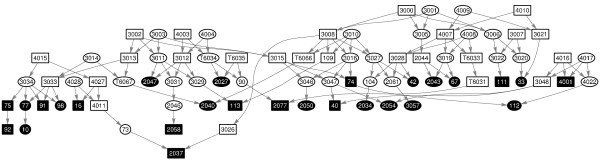
**Pedigree for a subset of FGFM family**. Dark nodes correspond to genotyped individuals. The complexity of the pedigree, together with the lack of information for how founders are related, makes parametric approaches for IBD detection unfeasible.

We used the algorithm to perform an analysis of genotype data obtained from 35 people, collectively identified as family FGFM, a large family known to be affected by an inherited form of kidney disease. These 35 individuals are all members of a large family whose self-reported pedigree has many uncertainties and missing links in the details of the relationships. In particular, the founders, as defined by the known pedigree, share a large number of IBD segments and as such they lack the property of genotype independence. Figure 8 shows just a small subset of this family.

Genetic data was collected from FGFM using the Affymetrix 100 k Array Set, with the goal of searching for an underlying genetic basis to the inherited form of kidney disease affecting many members of this family. The complexity of the pedigree, together with the lack of information for how founders are related, made parametric approaches unfeasible and was the motivating factor for developing an alternative methodology. We evaluate how the use of phased data improves the performance of our algorithm by first measuring how many loci are identified as IBD by the HMM with genotype emission and then using this information to compute phased genotype data. At this point, we iterate the HMM with phased genotype emission algorithm to identify IBD segments and to update the phase a few times.

For both HMM with genotype emission and with phased genotyped emission we used parameters *δ *= 40, *γ *= 8 and for the HMM with phased genotype emission we use *λ *= *μ *= 1/4. The average percentage of IBD detected by the two algorithms between two samples was, respectively, 14.14% and 16.36%. This increase, even if (as of yet) still insufficient to identify the disease-causing mutation in our case, was in fact dramatic, because it was mainly due to a whole new set of smaller IBD segments that we were now able to identify. This, was largely due to the fact that we were able to retrieve a great deal of the phase. In fact, for every sample, on average more than 90% of the loci were detected IBD with at least another sample of the family. This means that a given random consecutive pair of heterozygous loci has a good chance to encounter evidence to determine what their relative phase should be. This is in part due to the close relatedness of the people in the FGFM family and the high level of inbreeding.

## Discussion and Conclusions

We presented a new algorithm for the detection of IBD segments in genotype data. This approach uses the available phased genotype information to increase the accuracy of the detection, but at the same time is robust against inaccuracies of the phase. The method we present takes advantage of phase information even if this information is incomplete or less than fully accurate. The key components are (1) a new method for using phase information for the identification of IBD segments and (2) a new method for using IBD segment identification for the determination of phase. In one exemplary dataset derived from samples in a complex pedigree of a large complex family with a high rate of kidney disease (focal segmental glomerulosclerosis), this approach produced approximately 10% additional likely IBD loci compared with traditional methods. The ability to detect IBD segments, both long and short, will become increasingly critical for human genetic research, particularly in the search for genetic variants that are relatively rare and shared by only small subsets of people (as opposed to common variants that are present in large populations). It is therefore of paramount importance to be able to use the (necessarily) limited data available as best as possible in order to extract the full amount of information concealed in genotype data. When the fastest exact approaches [25,26] are not feasible, our algorithm can be a useful alternative. In fact, most exact algorithms have a computational burden exponential in the number of people in the pedigree. By contrast, our algorithm has a computational cost quadratic in the number of collected samples, requiring only that comparisons between all pairs of samples need to be performed. Our algorithm remains linear in the number of loci for which genotype data is available. It does not scale to the speed of [9], which is aimed at identifying short IBD segments with a computational cost that is linear in the number of people. However, unlike traditional approaches, our algorithm does not require precise pedigree information to run nor is it sensitive to pedigree loops, as are algorithms that perform exact inference (see [27]). Also, in our approach, there is no need to assume that the genotypes of the founders are independent, an assumption that can easily create significant problems, as can occur when two people distantly related share a long IBD segment.

This algorithm lends itself very well for iterative updates. For example, given a database of phased samples we can use this algorithm to perform phase inference on new samples using the IBD segments they will share with the samples already in the database. This would be very useful for the analysis of large biobanks of genetic data. The idea of using IBD segments to perform long-range phasing on genotype data from biobanks was introduced first in [14]. Our approach is different from this, perhaps conceptually simpler, and more robust. Most importantly, it is not strongly tied to the availability of correct pedigree data, which is often available only on a small scale, that is, only for nuclear subfamilies in the most recent generations. Moreover, it can take advantage of smaller IBD segment, in case larger ones are not available to phase at a given loci. We anticipate that this is but one of a number of future applications and venues for extension of this work.

## Authors' contributions

GG, GL, DR, and MP conceived the study and participated in its design. GG conceived the algorithms and performed the in silico experiments. GG, DR, and MP wrote the manuscript, which was edited by all authors. GL helped with the design of the algorithms and provided important suggestions. All authors read and approved the final manuscript.

## Appendix A - Analysis of HMM with haplotype and genotype emission

Given g a pair of observed genotypes for two samples at the same locus, define the variable

The possible values for lev_*G *_are shown in the last column of part A of Table 1. Think of lev_*G *_as the log-evidence in favor of being in state NO IBD. Approximately, if the overall log-evidence for a segment is smaller than -2*δ*, then the model would decode that segment as IBD, since it would be more likely to have switched at the beginning and at the end of the two segment than having remained in the NO IBD state. Notice that the probability of observing genotypes {AA, BB} is 0 for state IBD, since this state is incompatible with the locus observed being IBD, and therefore the value lev_*G*_({*AA*, *BB*}) would be infinite. In practice, this is weighted by the fact that we might believe that the locus has been genotyped incorrectly and therefore it is not absolute evidence to discard the hypothesis that the locus is in the IBD state. As a consequence, we modify lev_*G*_({*AA*, *BB*}) to be equal to a fixed constant *γ*.

If we were observing two haplotypes and we were trying to decide if they share an IBD segment, we would use a simpler model that we call *HMM with haplotype emission *and whose log-evidence we denote lev_*H*_. As before, given an observed pair of haplotypes for two samples at the same locus, *h*, we define

In part B of Table 1 we show the possible observed haplotypes together with their emission probabilities and the values for lev_*H*_.

Define the following random variables

where *g *is one of the six observable pair of genotypes in part A of Table 1, and *h *is one of the six observable pair of haplotypes in part B Table 1. The variables Σ_*IBD*||*NO IBD *_and Σ_*NO IBD*||*IBD *_represent, respectively, the log evidence pro IBD once the underlying Markov chain is in NO IBD state, and the log evidence pro NO IBD while the underlying Markov chain is in IBD state.

**Figure 9:**
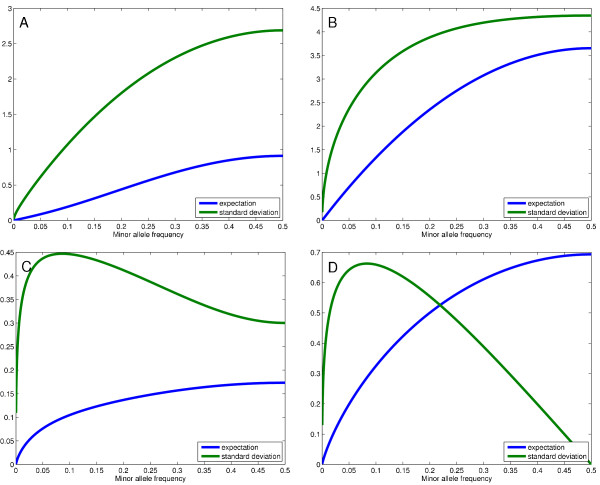
**Log-evidence comparison**. Expectation and standard deviation of random variable Σ_*IBD*||*NO IBD *_(A), Ω_*IBD*||*NO IBD *_(B), Σ_*NO IBD*||*IBD *_(C), and Ω_*NO IBD*||*IBD *_(D) as minor allele frequency varies (*γ *= 8).

The graph in Figure 9A shows expectation variance for Σ_*IBD*||*NO IBD *_and Figure 9B for Ω_*IBD*||*NO IBD *_as the minor allele frequency *p *for the allele at the observed locus varies between 0 and 1/2 and for *γ *= 8. The effective risk to fail to tag a segment as IBD when it is IBD is strictly related to the allele frequencies of the loci in the segment.

The ratio between expectation and standard deviation is clearly much smaller for Ω_*IBD*||*NO IBD *_than it is for Σ_*IBD*||*NO IBD*_, even more so when the minor allele frequency is large. Naively, alleles with large minor allele frequency are more likely to witness the fact that two segments are not IBD, even more so if we have available the haplotype rather than the genotype.

If a short sequence of loci is IBD, the HMM algorithm will recognize it as such if the collected log-evidence lev_*G *_or lev_*H *_will reach a threshold depending on the transition probabilities. Consider the HMM with genotype emission using the emission probabilities as in part A of Table 1. Figure 9C shows the expected value of Σ_*NO IBD*||*IBD*_, with respect to the allele frequency of that locus, together with the standard deviation. Notice that for alleles for which the minor allele frequency is very small, the standard deviation is very high. This is explained by the paradoxical fact that the log-evidence lev_*G *_can be positive, that is, in favor of state NO IBD, both for state {AA, BB} and one of the states {AA, AB} or {AB, BB}, despite the fact that either of these states are compatible with IBD state.

Using the model with the emission probabilities as in part B of Table 1, we get instead the graph in Figure 9D. The fact that the standard deviation for Σ_*NO IBD*||*IBD *_and Ω_*NO IBD*||*IBD *_is large when the minor allele frequency is small reflects the fact that the evidence for IBD might be very small if the allele shared IBD is the common one and huge if the observed allele is the rare one, so there is a wide range of possibilities. Again, the smaller ratio between expectation and standard deviation measures how much more unlikely we are to incur false negatives in detecting IBD segments when using haplotype data rather than genotype data. To better quantify this concept, we show some experimental results with data generated in silico in the Experiments section of this paper.

## Appendix B - Analysis of HMM with phased genotype emission

We will show in this appendix that the HMM with genotype is a particular case of the HMM with phased genotype when *λ *= *μ *= 1/2. Also, the HMM with haplotype is a particular case of the HMM with haplotype when *λ *= *μ *= 0, which implies that no transition among different IBD states is allowed. This shows intuitively how the HMM with phased genotype is a model which is at the same time flexible and precise.

**Figure 10:**
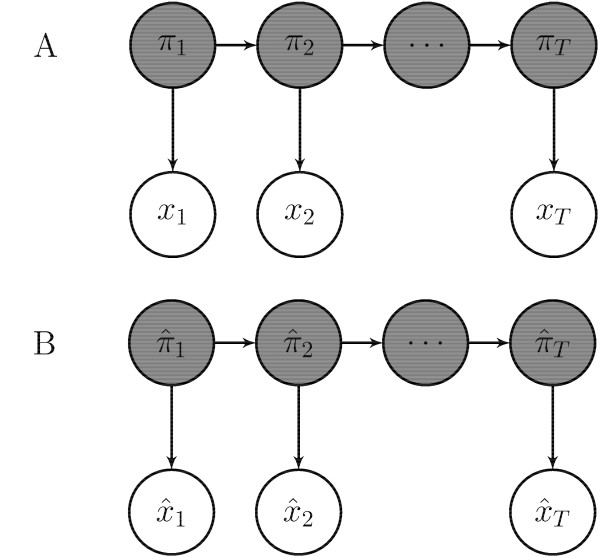
**Notation for HMMs**. Shaded nodes represent hidden states while unshaded nodes represent observed states for HMM with phased genotype emission (A) and HMM with genotype emission (B).

Following the notation in [[15], chap. 3], let {*π*_1_,...,*π*_*T*_} and {*x*_1_,...,*x*_*T*_} denote respectively the hidden and observed states for the HMM with phased genotype emission and {} and {} the corresponding ones for the HMM with genotype emission, as shown in Figure 10A-B. Notice that *x*_1_,...,*x*_*T *_correspond to the phased genotype and depend on the particular phase configuration chosen while  correspond to the genotype and are therefore independent of the phase. Define the function *ϕ *as the function outputs the corresponding hidden state for the HMM with genotype emission given a hidden state for the HMM with phased genotype emission. For example *ϕ*(LL) = IBD and *ϕ*(NO IBD) = NO.

**Lemma 1 **If we choose coefficients *λ *= *μ *= 1/2, then for any hidden states *i *and , the following holds(2)

where  is chosen according to the following table.

Notice that  equals the number of observed states *x*_*t*+1 _corresponding to the observed state .

**Proof **Using part A of Table 1 and Table 5, Lemma 1 follows by checking the different cases for hidden states *i*, , and observed states .

**Table 5:** HMM with phased genotype emissions.

Phased genotype	NO	LL	LR	RL	RR
(AA, AA)	*p*^4^	*p*^3^	*p*^3^	*p*^3^	*p*^3^
(AA, AB)	*p*^3^*q*	*p*^2^*q*	0	*p*^2^*q*	0
(AA, BA)	*p*^3^*q*	0	*p*^2^*q*	0	*p*^2^*q*
(AA, BB)	*p*^2^*q*^2^	0	0	0	0
(AB, AA)	*p*^3^*q*	*p*^2^*q*	*p*^2^*q*	0	0
(AB, AB)	*p*^2^*q*^2^	*pq*^2^	0	0	*p*^2^*q*
(AB, BA)	*p*^2^*q*^2^	0	*pq*^2^	*p*^2^*q*	0
(AB, BB)	*pq*^3^	0	0	*pq*^2^	*pq*^2^
(BA, AA)	*p*^3^*q*	0	0	*p*^2^*q*	*p*^2^*q*
(BA, AB)	*p*^2^*q*^2^	0	*p*^2^*q*	*pq*^2^	0
(BA, BA)	*p*^2^*q*^2^	*p*^2^*q*	0	0	*pq*^2^
(BA, BB)	*pq*^3^	*pq*^2^	*pq*^2^	0	0
(BB, AA)	*p*^2^*q*^2^	0	0	0	0
(BB, AB)	*pq*^3^	0	*pq*^2^	0	*pq*^2^
(BB, BA)	*pq*^3^	*pq*^2^	0	*pq*^2^	0
(BB, BB)	*q*^4^	*q*^3^	*q*^3^	*q*^3^	*q*^3^

The matrix displays emission probabilities for hidden states NO, LL, LR, RL, RR for the HMM with genotype emission, with *p *the minor allele frequency, and *q *= 1 - *p*.

**Lemma 2 **If we choose coefficients *λ *= *μ *= 1/2, then for any hidden states *i *and , the following holds(3)

**Proof **To prove the equation (3), we first use Bayes theorem to get

Using equation (2) we continue to derive

**Proposition 1 **If we choose coefficients *λ *= *μ *= 1/2, then for any hidden states *i *and ,(4)

and(5)

**Proof **We prove the proposition by induction. Suppose the statement (4) is true for *t *= *n*. Then

where the second equation follows from equation (3).

We use induction in the reverse order for the statement in (5). We start by

and then assuming the statement true for *t *= *n *and using equation (2),

**Theorem 1 **If we choose coefficients *λ *= *μ *= 1/2, then for any hidden state 

**Proof **By applying Bayes theorem and decomposing the expression on the left hand and using equations (5) and (4)

Because of the previous statement, using coefficients *λ *= *μ *= 1/2 will give as a result that the probability for being in one of the four IBD states for the HMM with phased genotype emission is the same as the probability for being in IBD state according to the HMM with genotype emission. Therefore it will not increase our ability to detect IBD segments. Although using coefficients *λ *= *μ *= 0 will indeed increase our ability since the costs of switching back and forth from NO IBD state to one of the IBD states are about the same for the HMM with haplotype emission and the HMM with phased genotype emission, other than for a small factor of 4, but we would need to have accurate phased genotype data. The nice property of the HMM with phased genotype emission is that we can tune the parameters *λ *and *μ *to some intermediate values, so that we allow for inaccurate phase but we still take advantage of it to detect small IBD segments. A choice of *λ *= *μ *= 1/4 would for example increase the ability to detect smaller IBD segments and still be very robust against errors in the phase.

## Appendix C - Link and phase update pseudocode

### Algorithm 1 - Link update algorithm

Given a set of phased genotypes, this algorithm creates a matrix of links for each phased genotype that will be later used to perform phase update. The algorithm will identify all IBD segments among each pair of samples and for each individual it will update a sparse upper triangular matrix with the links generated by each detected IBD segment.

**Input: **Phased genotype {*G*_1_, *G*_2_,...,*G*_*n*_} from *n *samples.

**for all **pair of samples **do**

   Identify IBD segments in between the two samples using the HMM with phased genotype.

   **for all **IBD segments detected **do**

      Define one sample as the *source *and one sample as the *target*.

      Identify loci *t*_1 _< ... <*t*_*m*_for which the *target *genotype is heterozygous.

      Identify indexes *i*_1 _< ... <*i*_*k *_for which the *source *phased genotype at loci  is homozygous AA or BB and the *target *phased genotype is heterozygous AB or BA for *j *= 1,...,*k*.

      **for ***j *:= 1 to *k *- 1 - **do**

         **if ***G*_*source*_() = *G*_*source*_() **then**

            *L*_*target*_(*i*_*j*_, *i*_*j*+1_) := *L*_*target*_(*i*_*j*_, *i*_*j*+1_) + 1

         **else**

            *L*_*target*_(*i*_*j*_, *i*_*j*+1_) := *L*_*target*_(*i*_*j*_, *i*_*j*+1_) - 1

         **end if**

      **end for**

      Swap *target *and *source *and repeat once.

   **end for**

end for

**return **Sparse matrices {*L*_1_, *L*_2_,...,*L*_*n*_}.

### Algorithm 2 - Phase update algorithm

Given a phased genotype with *m *heterozygous loci and phase choice represented by an *m *× 1 binary vector, an *m *× *m *sparse matrix of links *L*, this algorithm generates a new choice for the phase. The idea is to identify first for every consecutive heterozygous loci which links are affected by modifying the relative phase in between the two. Then, for every sliding window of a given length *s*, all possible combinations of phases are tested to identify which one satisfies the maximum number of links. Coefficients *z*_*i *_represent the present relative phase between the *i*-th and *i *+ 1-th heterozygous locus. For every nonzero entry in *L *the sparse matrix *B *keeps track of all the relative phase coefficients which determine if the corresponding links are satisfied or not while the vector *S *keeps track of the amount of links for each nonzero entry of *L*. In the second part of the algorithm, using a sliding window of length *s *all possible configurations for the relative phase coefficients withing that sliding window are tested and the one which maximizes the amount of links is chosen. Whenever a change is made, a *flag *variable is raised, and the process reiterates until no *flag *variable is raised, that is, no further changes within a sliding window of length *s *can increase the amount of satisfied links.

**Input: **Current *m *× 1 phase binary vector *Y *= (*y*_1_, *y*_2_,...,*y*_*m*_).

**Input: **Sparse *m *× *m *matrix *L *with link coefficients collected from IBD segments.

**Input: **Window length *s*.

Initialize *r *× *m *binary sparse matrix *B *with *r *the number of nonzero entries of *L*, *r *× 1 vector *S*, and *k *:= 1.

Compute coefficients *z*_*i *_:= *y*_*i *_⊕ *y*_*i*+1 _for *i *= 1,...,*m *- 1.

**for all **nonzero entries in *L *at position *i*,*j ***do**

   **for ***l *:= *i *to *j *- 1 **do**

      *B*(*k*, *l*) := 1.

   **end for**

   **if ***y*_*i *_= *y*_*j *_**then**

      *S*(*k*) := *L*(*i*, *j*).

   **else**

      *S*(*k*) := - *L*(*I, j*).

   **end if**

   *k *:= *k *+ 1.

end for

Initialize *flag *:= 1.

**while ***flag *= 1 **do**

   *flag *:= 0.

   **for ***i *:= 1 to *m *- 1 **do**

      **for all **binary vectors (*c*_0_, *c*_1_, *c*_2_,...,*c*_*s*_) with *c*_0 _= 1 **do**

         Compute vector 

         **if ****then**

            *z*_*i*+*k *_:= *z*_*i*+*k *_⊕ *c*_*k *_for *k *= 0,...,*s*

            *S*(*k*) := - *S*(*k*) for *k *such that *w*(*k*) = 1

            *flag *:= 1.

         **end if**

      **end for**

   **end for**

end while

Recompute coefficients *y*_*i*+1 _:= *y*_*i *_⊕ *z*_*i *_for *i *= 1,...,*m *- 1.

**return **updated phase binary vector *Y *= (*y*_1_, *y*_2_,...,*y*_*m*_).
